# 514. Nirmatrelvir/ritonavir or Molnupiravir for Treatment of Non-hospitalized Patients with COVID-19 at Risk of Disease Progression

**DOI:** 10.1093/ofid/ofad500.583

**Published:** 2023-11-27

**Authors:** Adeel A Butt, Peng Yan, Obaid Shaikh

**Affiliations:** VA Pittsburgh Healthcare System, Pittsburgh, PA; VA Pittsburgh Healthcare System, Pittsburgh, PA; VA Pittsburgh Healthcare System, Pittsburgh, PA

## Abstract

**Background:**

In randomized controlled trials, Nirmatrelvir/ritonavir (NMV/r) and Molnupiravir (MPV) reduced the risk of severe/fatal COVID-19 disease. Real-world data are limited, particularly studies directly comparing the two agents.

**Methods:**

Using the VA National COVID-19 database, we identified previously uninfected, non-hospitalized individuals with COVID-19 with >1 risk factor for disease progression who were prescribed either NMV/r or MPV within 3 days of a positive test. We used inverse probability of treatment weights (IPTW) to account for providers’ preferences for a specific treatment. Absolute risk difference (ARD) with 95% confidence intervals were determined for those treated with NMV/r vs. MPV. The primary outcome was severe, critical, or fatal disease within 30 days of treatment prescription using the IPTW approach. Analyses were repeated using propensity-score matched groups and 1:1 matched groups.

Cohort construction

**Figure:**
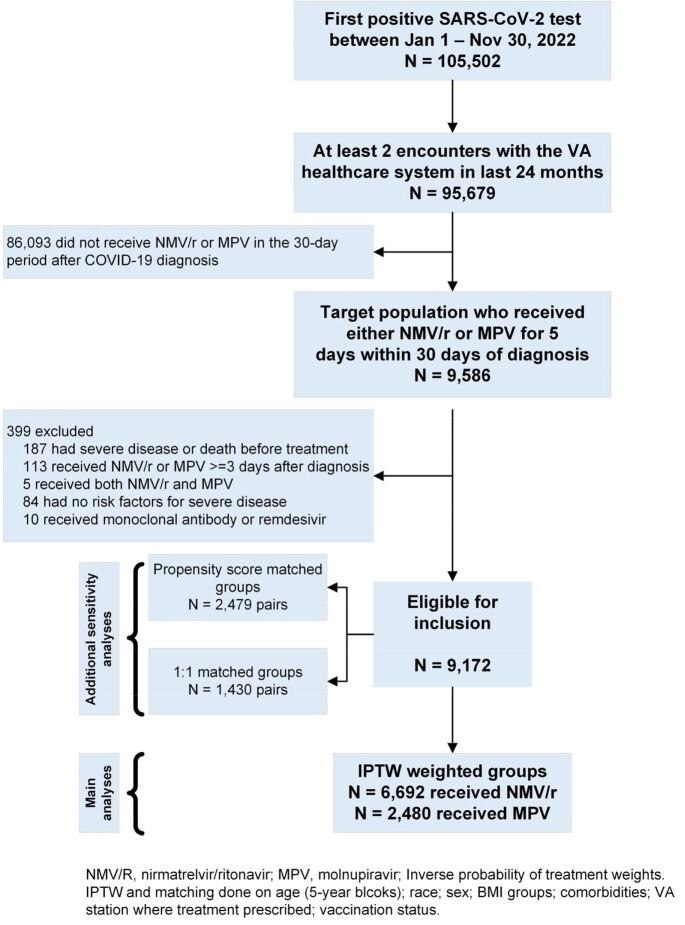

**Results:**

Between January 1 and November 30, 2022, 9,172 individuals were eligible for inclusion (6,692 prescribed NMV/r; 2,480 prescribed MPV). The ARD for severe, critical, or fatal disease for NMV/r vs MPV was -0.18 (95% CI -0.45 to 0.09). There was no statistically significant difference in ARD among strata by age, race, sex, comorbidities, or symptoms at baseline. Kaplan-Meier curves did not demonstrate a difference between the two groups (p-value=0.43). Analysis of the propensity-score matched cohort yielded similar results (ARD for NMV/r vs. MPV -0.12, 95% CI -0.71 to 0.47).

**Figure:**
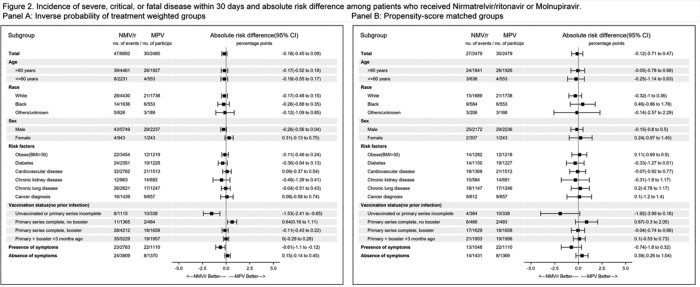

**Figure:**
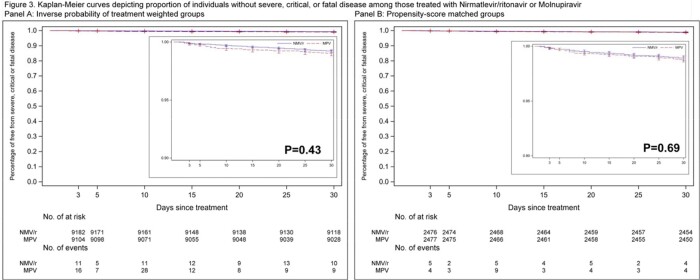

**Conclusion:**

We found no significant difference in short term risk of severe, critical or fatal disease in non-hospitalized individuals with COVID-19 at risk of disease progression treated with either NMV/r or MPV.

**Disclosures:**

**Adeel A. Butt, MBBS, MS**, Gilead Sciences: Grant/Research Support|Merck and Company: Grant/Research Support

